# Current Status of Peri-Implant Diseases: A Clinical Review for Evidence-Based Decision Making

**DOI:** 10.3390/jfb14040210

**Published:** 2023-04-10

**Authors:** Antonio Scarano, Ahmad G. A. Khater, Sergio Alexandre Gehrke, Paola Serra, Inchingolo Francesco, Mariastella Di Carmine, Sergio Rexhep Tari, Lucia Leo, Felice Lorusso

**Affiliations:** 1 Department of Innovative Technologies in Medicine and Dentistry, University of Chieti–Pescara, 66100 Chieti, Italy; 2 Health Affairs Directorate, Egyptian Ministry of Health and Population, Banisuif 62511, Egypt; 3 Department of Research, Bioface/PgO/UCAM, Calle Cuareim 1483, Montevideo 11100, Uruguay; 4 Department of Biotecnology, Universidad Católica de Murcia (UCAM), 30107 Murcia, Spain; 5 Department of Interdisciplinary Medicine, Section of Dental Medicine, University of Bari “Aldo Moro”, 70124 Bari, Italy

**Keywords:** dental implant, evidence-based practice, mucositis, peri-implant disease, peri-implant health, peri-implantitis, periodontal disease, review

## Abstract

Background: the prevalence of peri-implant diseases is constantly growing, particularly with the increasing use of dental implants. As such, achieving healthy peri-implant tissues has become a key challenge in implant dentistry since it considers the optimal success paradigm. This narrative review aims to highlight the current concepts regarding the disease and summarize the available evidence on treatment approaches clarifying their indications for usage following the World Workshop on the Classification of Periodontal and Peri-implant Diseases (2017). Methods: we reviewed the recent literature and conducted a narrative synthesis of the available evidence on peri-implant diseases. Results: scientific evidence on case definitions, epidemiology, risk factors, microbiological profile, prevention, and treatment approaches for peri-implant diseases were summarized and reported. Conclusions: although there are numerous protocols for managing peri-implant diseases, they are diverse and nonstandardized, with no consensus on the most effective, leading to treatment confusion.

## 1. Introduction

Dental implant-supported prostheses are a well-established rehabilitation treatment for partially or completely edentulous patients that restore function and esthetics while having long-term survival rates [1]. As a result, the use of current treatment protocols for rehabitulating edentulous patients, including those with severe bone deficiency, is increasing, which is tied to a rise in the incidence of peri-implant diseases. Although there are various protocols for managing peri-implant diseases, the treatment is complex and nonstandardized, and new techniques still need to be investigated. Therefore, we conducted this narrative review to assist clinicians and surgeons in making decisions by summarizing the most recent disease and clinical recommendations presented by the World Workshop on the Classification of Periodontal and Peri-implant Diseases (2017) [2]. The tissues surrounding osseointegrated dental implants are referred to as peri-implant tissues, consisting of soft and hard tissue parts. The soft tissue part forms following the placement of implant/abutment during the wound healing and is known as “peri-implant mucosa,” while the hard tissue part makes contact with the implant surface to ensure implant stability. As such, the new classification differentiated peri-implant diseases into peri-implant health (the optimum), peri-implant mucositis (soft tissue part), and peri-implantitis (hard tissue part).

## 2. Peri-Implant Diseases and Conditions

### 2.1. Peri-Implant Health

Healthy peri-implant tissues is a more objective definition of implant success that focuses on the biological and esthetic health of surrounding tissues in addition to the implant function rather than just implant survival. This is clinically assessed by:

- Absence of clinical inflammatory signs;
- No bleeding and suppuration on mild probing (0.25 N);
- Stable probing depth compared to previous visits;
- Absence of radiographic bone loss (excluding physiological crestal bone loss one year after the prosthetic load of 0.5–2 mm) [3].

The physiological probing depth around implants cannot be determined since the absence of the periodontal ligament provides less resistance to probe insertion. Consequently, excessive force on probing can disrupt the integrity of the mucosa-abutment attachment and produce bleeding on healthy implants. As such, we could have peri-implant health on implants with reduced bone support, successfully treated implants, or implants positioned in a diminished alveolar crest [4]. 

**Figure 1:**
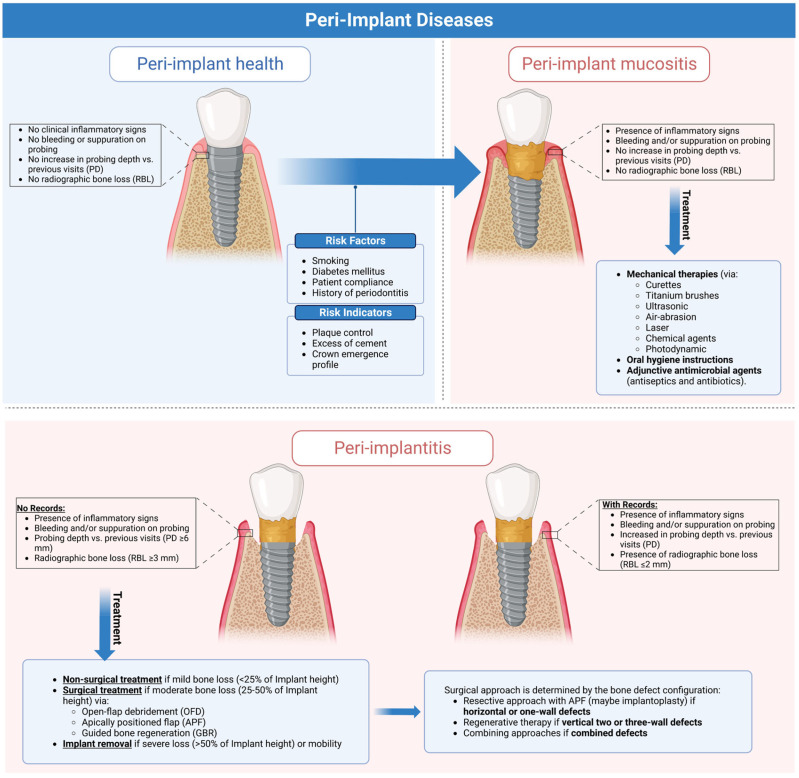
Graphical abstract illustrating the clinical characteristics of peri-implant health and diseases and current evidence-based approaches for managing peri-implant diseases. This figure was created using Biorender.com (https://app.biorender.com).

The peri-implant mucosa section facing the implant consists of two compartments, a “coronal” compartment lined with the sulcular epithelium and a thin epithelial attachment, whereas the “apical” compartment contacts the connective tissue implant surface. Histologically, this mucosa contains a connective tissue core, often covered by orthokeratinized epithelium on its outer surface [3]. Therefore, after abutment placement, the mucosal height gradually stabilizes to 3–4 mm, with a 2 mm epithelium [5] (Figure 1 and Table 1).

### 2.2. Peri-Implant Mucositis

**Figure 2:**
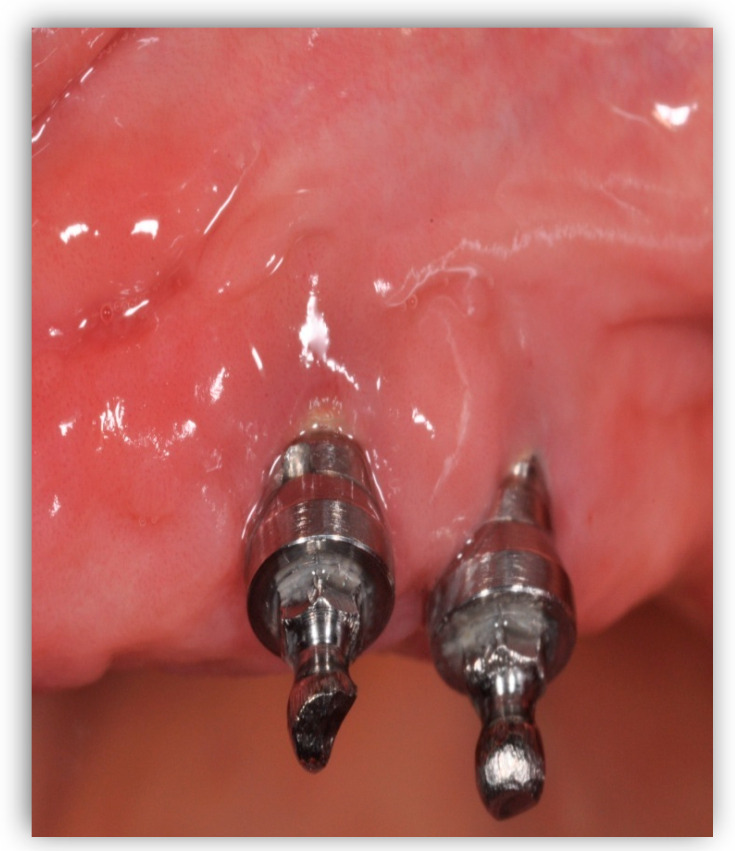
Peri-implant mucositis; there is gingival inflammation around the implant unit.

Peri-implant mucositis (Figure 2) is an inflammation in the peri-implant tissue that is clinically identified by bleeding and/or suppuration on probing without radiographic bone loss [6].

**Figure 3:**
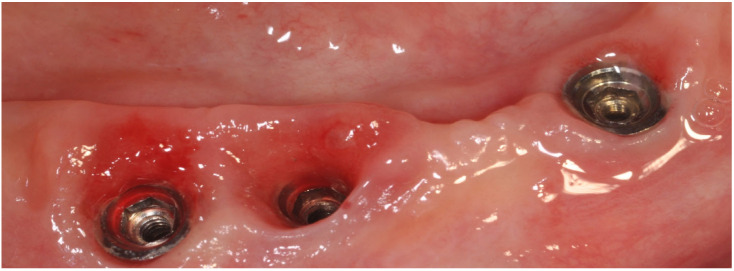
Peri-implant mucositis; inflammatory tissues induced by abutment loosening.

Although a recognized cause–effect relationship exists between mucositis and plaque accumulation [7], reestablishing good oral hygiene could restore the clinical parameters and biochemical markers in the peri-implant crevicular fluid to normal. Even if implants have less plaque accumulation, they often show more inflammation and bleeding sites than teeth [8]. Histologically, the inflammatory lesion is found in the connective tissue, lateral to the epithelial barrier, and it is more extensive for advanced lesions (0.36 mm^2^) than in the early lesion (three weeks 0.14 mm^2^) (Figure 3).

### 2.3. Peri-Implantitis

**Figure 4:**
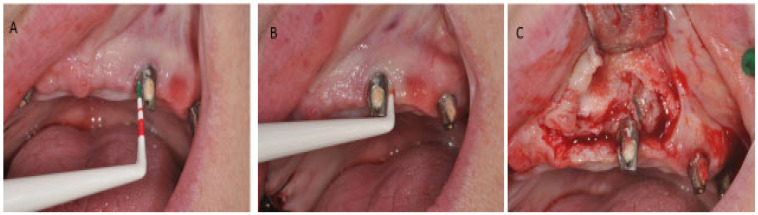
Peri-implantitis; (**A**) Clinical signs. (**B**) Probing depth. (**C**) Peri-implant inflammatory tissue.

Peri-implant mucositis is an inflammation in the peri-implant soft tissues with a progressive loss in the supporting bone. It is clinically identified by bleeding and/or suppuration on probing, increased pocket depth compared to previous visits, and radiographic bone loss [9] (Figure 4).

**Figure 5:**
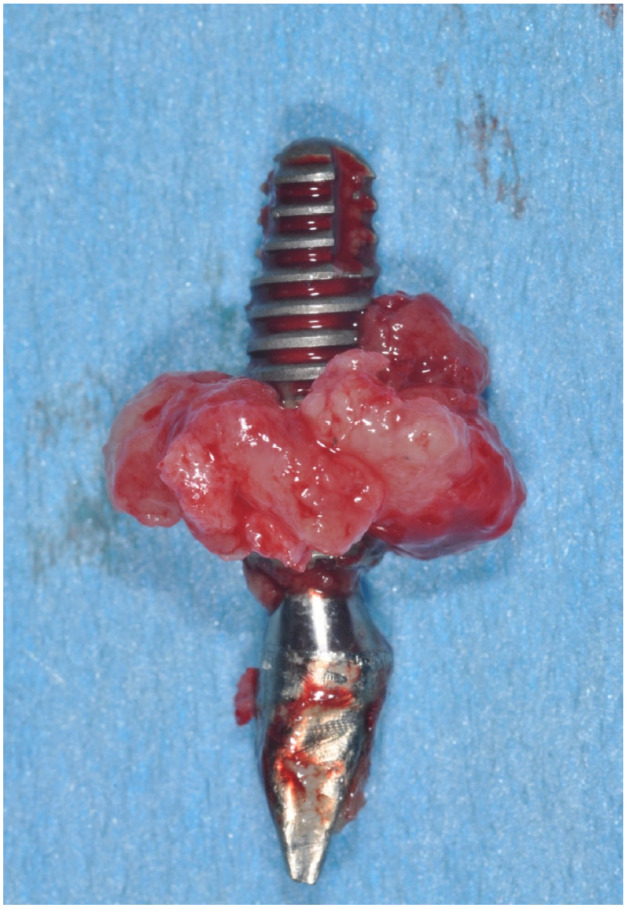
Clinical aspect of implant removed for peri-implantitis.

Standardized intraoral radiographs should be performed to monitor changes in bone levels around implants beginning with abutment placement, as crestal losses of more than 2 mm (physiological remodeling) from the baseline should be considered pathological [4]. When previous clinical or radiographic data are unavailable, the diagnosis should be confirmed by bleeding and/or suppuration on probing, a pocket depth greater than 6 mm, and more than 3 mm of radiographic bone loss (measured from the most coronal part of the marginal bone crest). Histologically, the inflammation becomes twice the size of the periodontal lesion (3.5 mm^2^ versus 1.5 mm^2^), extending to the bone crest and containing more neutrophils and lymphocytes B CD19+ than mucositis and more polymorphonuclear and macrophages than periodontitis [10,11] (Figure 5).

However, Derks et al. [12] found that initial radiographic bone loss occurs between the second and third functional years. Thus, peri-implantitis develops early, and bone loss progresses in a nonlinear and accelerated pattern with significant individual variability. According to Fransson et al. [13], in a retrospective study, implants had an average bone loss of 1.7 mm after one year, and implants with radiographic bone loss of more than 1, 2, and 3 mm were 68%, 32%, and 10%, respectively.

## 3. Epidemiology of Peri-Implant Diseases

Evaluation of the diseases’ epidemiological features aims to determine how frequently and why the disease arises. Such epidemiological data are used to assess and improve available disease-specific prevention approaches, which are often implemented to target the affected population [14]. As such, Derks et al. showed that the prevalence of peri-implant mucositis ranged from 19% to 65% and peri-implantitis from 1% to 47% [15]. However, Lee et al., in a meta-analysis, showed that the mean prevalence of peri-implant mucositis was 29.48% (implant-based) and 46.83% (subject-based), and the mean prevalence of peri-implantitis was 9.25% (implant-based) and 19.83% (subject-based) [16]. Moreover, Rakic et al. revealed in a meta-analysis that the peri-implantitis prevalence was 18.5% (implant-level) and 12.8% (patient-level) [17]; however, Dreyer et al. revealed in a meta-analysis that the peri-implantitis prevalence ranged from 1.1% to 85.0% (implant-level) and an incidence ranged from 0.4% (within three years) to 43.9% (within five years) [18].

Such variations in prevalence are due to methodological heterogeneity in reporting the peri-implant biological complications in studies, which limits attempts to estimate the true impact of peri-implant diseases globally; thus, that emphasizes the importance of fully adopting case definitions by the World Workshop on the Classification of Periodontal and Peri-implant Diseases (2017) for accurate prevalence estimation [19].

## 4. Risk Factors and Indicators

Risk factors are disease-causing agents usually established by longitudinal research, whereas risk indicators depend on cross-sectional investigations [20]. We summarized several risk factors and risk indicators of peri-implant diseases in Figure 3. Smoking and diabetes are the most well-known systemic risk factors that have been reliably related to peri-implant disorders. The odds ratio (OR) of smoking was 2.7 in the study by Schwarz et al. [9], 31.6 in Rinke [21], 4.67 in Roos Jansaker et al. [22], while the OR of diabetes was 2.5 in Dreyer et al. [18], and Monje et al. [23] found that diabetic patients had about a 50% higher risk of peri-implantitis than nondiabetics (OR = 1.89). On the other side, there was a strong correlation between peri-implantitis and periodontitis history (OR ranging from 2.2 to 9.2) [9], as evidenced by a lower frequency of bone loss (>3 mm) in healthy individuals (5%) versus patients with moderate periodontitis (11%) and severe periodontitis (15%) [24].

Poor plaque control is a risk factor since it makes it difficult to reach the implant sites during oral hygiene, explaining the higher incidence of peri-implantitis in areas with limited access to oral hygiene (65%) compared to cleanable areas (18%) [25]. Excess cement retained around peri-implant soft tissues promotes plaque accumulation due to its rough surface [26], and its removal promotes the normalization of inflammation indices [27]. Thus, cemented prostheses without cement residues appear to have no increased risk of developing peri-implantitis compared to screw-retained prostheses (Figure 4). Adherence to oral hygiene instructions is also a risk factor, as Costa et al. [28] and Roccuzzo et al. [24] found that individuals who followed a regular maintenance program had a lower incidence of peri-implantitis (18% and 27%, respectively) than those who did not (43% and 47%). There is controversy about the minimum amount of keratinized mucosa around the implant; however, many observational studies indicated that keratinized mucosal deficiency (<2 mm) causes more discomfort in oral hygiene procedures, higher plaque levels, deeper pockets, and bleeding [29,30]. 

**Figure 6:**
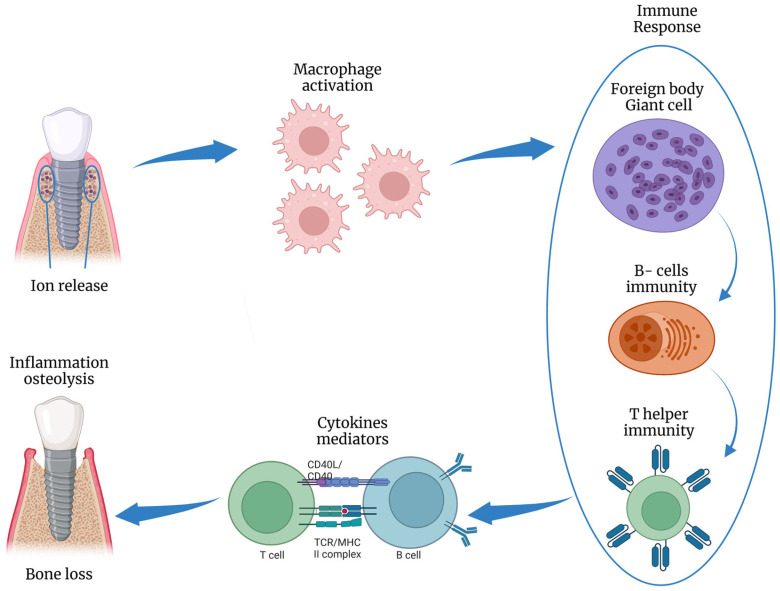
Schematic illustration of the inflammatory response to titanium ions release, inducing osteolysis. This figure was created using Biorender.com.

The presence of peri-implantitis has been associated with many gene polymorphisms (e.g., IL-1 gene) and osteoprotegerin (IL-6, and TNF7). In addition, Titanium and/or iron particles were identified in peri-implant tissue biopsies and inflammatory cells at peri-implantitis areas [31]. Titanium atoms may boost T-lymphocyte differentiation towards the osteoclastic pathway [32] and could alter microbial genetic expression via epigenetic DNA methylation [33] (Figure 6).

However, iatrogenic factors that dental professionals generate, such as too buccal or too apical position (>6 mm to the enamel–cement junction of the adjacent tooth), incorrect abutment placement, and regenerative procedures, may play a role in the etiology of peri-implantitis [34]. Although the literature has not yet emphasized occlusal overload as a risk factor for peri-implantitis, a regular assessment of patients’ occlusion is recommended. Also, the gap between the implant and the abutment could act as a bacterial reservoir, causing chronic inflammation and marginal bone loss [35]; hence, tissue-level implants should be introduced to move this gap away from the bone crest, or antibacterial coating should be applied to the system's internal chamber [36,37].

## 5. Microbiological Profile

Given that peri-implant diseases are induced by an inflammatory process around the implant, which can impact soft tissue or progress to hard tissue, resulting in bone loss. Thus, identifying the causative triggers is critical in understanding disease progression and management [38]. Although the microbiota in healthy peri-implant tissue is similar to the healthy periodontium [39,40], the periodontopathogenic microorganisms in periodontitis around natural teeth differ from pathogens associated with diseased implant sites [41,42,43]. *Porphyromonas gingivalis*, *Prevotella intermedius/nigrescens*, and Uncultivable asaccharolytic anaerobic bacteria (Gram-positive and -negative rods) often colonized peri-implantitis sites, whereas the opportunistic microorganisms were not frequently identified [44].

Compared to peri-implant health, some specific bacterial species (*Campylobacter rectus*, *Campylobacter gracilis*, *Treponema denticola*, *Tannerella forsythia*, *Treponema socranskii*, *Porphyromonas gingivalis*, *Prevotella intermedia*, and *Staphylococcus aureus*) were substantially associated with peri-implant lesions [45]. Also, *Candida* spp. and fungal species were frequently identified in peri-implant lesions [46], indicating that such microbial colonization may contribute to the onset of peri-implant disease [43]. Moreover, a clinical study revealed a significant correlation between viruses (particularly human cytomegalovirus and the Epstein–Barr virus) and clinical parameters of peri-implant diseases since HCMV and EBV were highly prevalent in subgingival plaque of peri-implantitis, further suggesting the potential role of viruses in peri-implant disease progression [47].

## 6. Prevention of Peri-Implant Diseases

With the increasing prevalence of peri-implantitis, and its irreversible condition with limited and expensive treatments, preventing peri-implant diseases is becoming paramount to decreasing its incidence and boosting implant success rates [48,49]. As such, The European Federation of Periodontology (EFP) established some recommendations for managing the primary risk factors of peri-implant diseases throughout implant workflow [48]. Proper personalized risk assessment for the individual patient is the primary key to these preventive measures, which aim to address and modify any relevant risk factors (either local or systemic) [49]. 

Such preventive measures begin before implant placement (“Primordial prevention”) by addressing the underlying risk factors that may induce disease development (e.g., preventing noncommunicable diseases (diabetes type-II) by healthy behavior promotion such as not smoking, increasing physical activity, and healthy diets) [49]. Once implant placement occurs, preventive measures (“Primary prevention”) are implemented to maintain the peri-implant tissues healthy over time and address any risk factors that may trigger disease onset, such as regularly controlling biofilm accumulation around implants and practically educating and motivating patients on oral hygiene measures). Then, early management and control of peri-implant mucositis should be implemented to prevent peri-implantitis progression (“Secondary prevention”) [49].

Currently, there is no direct evidence evaluating the impact of primordial preventive (i.e., pre-implant placement) or primary preventive (i.e., post-implant placement) interventions on the development and progression of peri-implant diseases [50]. However, in Carra et al., a meta-analysis revealed limited evidence supporting glycaemic control in diabetic patients and regular supportive periodontal/peri-implant care to prevent peri-implantitis development. Also, augmentation procedures performed at implant sites with peri-implant keratinized mucosa deficiency may reduce peri-implant inflammation and marginal bone loss [50].

## 7. Treatment Strategies

Although there are various protocols for managing peri-implant diseases, they are variable and nonstandardized, with no consensus on the most effective, creating confusion in the treatment. Therefore, we aimed to highlight the current concepts in treatment approaches and the available evidence on their efficacy and clarify their indications for usage. Management of peri-implant diseases could be through nonsurgical therapy, surgical therapy, or implant removal, depending on the condition and clinical symptoms [50].

### 7.1. Nonsurgical Treatment

Nonsurgical therapy includes mechanical debridement, oral hygiene instructions, and possibly local antiseptics, which are indicated in managing mucositis and peri-implantitis with mild bone loss (<25% of implant height) [51]. Mechanical debridement of the implant surface aimed to reduce the adhered biofilm and restrict bacterial colonization to maintain peri-implant health. That debridement could be achieved by curettes ultrasonic instruments, titanium brushes, air power abrasion, laser, chemical agents, and photodynamic therapy [52,53]:

- Curettes plastic or carbon-fiber curettes are softer since they can mechanically debride the implant surface without damaging it. However, they cannot clean the spaces between the threads. Stainless steel curette is contraindicated since its hardness is greater than titanium, resulting in substantial damage to the implant surface [52,54];
- Ultrasonic Instruments: specialized ultrasonic tips that are implant compatible (a steel tip, Teflon tip, and tip covered with either polyether ketone PEEK or carbon fiber), vibrating at high frequencies (>20 kHz), can effectively debride the implant surface and remove subgingival tartar faster than manual instruments [54,55,56];
- Titanium Brush: these brushes are often used during open flap debridement. However, they are fragile and break easily;
- Air Powder Abrasion: granules of glycine, hydroxyapatite, sodium bicarbonate, titanium dioxide, erythritol, or tricalcium phosphate can be used to debride the implant surface without damaging it. However, these devices must be used cautiously (especially subgingivally) to avoid soft tissue injury or emphysema [57,58];
- Laser: many lasers can decontaminate the implant surface at high doses, and their capacity is dose dependent. It can irreversibly alter the implant surface; it is critical to consider proper time and emission power to avoid the possibility of thermal osteonecrosis of the bone. There is no clear evidence in the literature distinguishing between laser, mechanical treatment alone, combined with mechanical debridement, and air powder abrasion [59,60,61]. As such, Cosgarea et al. found insufficient evidence of the clinical efficacy and patient-reported benefits of mechanical/physical decontamination in nonsurgical submarginal instrumentation [19]. In all cases, inflammatory indices were reduced for a limited follow-up period (3–6 months), while longer periods were associated with reinfection phenomena [59];
- Photodynamic Therapy: a low-power laser light (usually diode) or a nonlaser infrared light stimulates the production of reactive oxygen species (ROS) from a photosensitive molecule (methylene blue and toluidine blue, porphyrins, chlorines, and phthalocyanines). Photodynamic therapy destroys bacteria directly and speeds tissue healing; it should be used as an adjunct to mechanical debridement or surgery. However, Ramanauskaite et al., in a systematic review, revealed limited data with inconclusive evidence on the clinical effectiveness of photo/mechanical and physical implant surface decontamination in conjunction with surgical peri-implantitis treatment [62]. Therefore, the literature has yet to prove that this treatment is superior to conventional decontamination procedures, and there is no consensus on the factors making this therapy more successful (type of light source, wavelength, and time of therapy), so additional research is needed [63];
- Chemical methods: chemical agents with antimicrobial effects (e.g., hydrogen peroxide, phosphoric acid, EDTA, and NaOCl) can efficiently debride the implant surface. However, these agents could alter the titanium surface’s integrity and produce chemical residues having a cytotoxic effect. Therefore, they should be used at a moderate concentration (citric acid 20%, EDTA 24%, NaOCl 1.5%) [64]. Yet, Dommisch et al., in a systematic review, concluded limited evidence that the adjunctive use of aPDT, 0.95% NaOCl, and 0.12% CHX had no additional efficacy in improving bleeding on probing or pocket depth as compared to submarginal instrumentation during peri-implant mucositis treatment [65]. Also, Wilensky et al., in a meta-analysis, found a low quality of evidence supporting not employing chemicals (PDT, CHX, and LAbs) for surface decontamination during peri-implantitis surgery since mechanical debridement (either with or without saline) was superior [66];
- Adjunctive Measures: such measures have been employed in response to the necessity to eliminate dental biofilm to maintain peri-implant tissues healthy regularly [53,66]; thus, adjunctive patient usage of some chemical agents (e.g., antimicrobials, anti-inflammatory, or probiotic drugs) after mechanical treatment of peri-implant diseases could aid in resolving inflammation around implants [67,68,69]. Antibiotics (systemic and local) have been shown to reduce bleeding on probing and pocket depth [67,68], and systematic antibiotics significantly improved the modified implant surface treatment when combined with mechanical debridement with no effect on nonmodified surfaces [69]. Gennai et al., concluded the current evidence as the systemic application of probiotics, antiseptics, and antibiotics for three months at least have clinical improvements by reducing gingival and plaque indices when combined with submarginal instrumentation for peri-implant mucositis patients [70]. However, Teughels et al. revealed insufficient evidence to support systemic and local antimicrobials as adjuncts during surgical treatment of peri-implantitis since they did not improve clinical outcomes (e.g., pocket depth, bleeding on probing, etc.) [71]. Given the inconclusive evidence on their efficacy and concerns about increasing antibiotics resistance or superinfection by opportunistic microorganisms [72], further randomized clinical trials are needed to validate their adjunctive usage;
- Electrochemical Disinfection: the main principles regarding this technique involve the surface disinfection of biofilm-contaminated implant surfaces that could take advantage of the application of low direct currents [73]. Recent studies reported that the current charge is effective for organic component removal of the oral biofilms and could reduce the adhesion capabilities and the oral environment survival [74,75]. For this purpose, Koch et al., evaluated boron-doped diamond (BDD) electrodes (>2.5 V) to determine an electrochemical implant surface treatment against biofilms producing a water electrolytic property with hydrogen release from the cathode and oxygen from the anode [73].

In summary, nonsurgical treatment is effective in treating mucositis, whereas it is the initial stage for managing peri-implantitis to achieve healthier soft tissues before undergoing surgical therapy. Only limited and unpredictable improvements of the main clinical and inflammatory parameters (particularly BOP reduction) have been reported, with a clear tendency to reinfection. The greatest reduction in pocket depth was 1.2 mm, with no bone gain [76].

### 7.2. Surgical Treatment

Surgical therapy is often recommended for treating peri-implantitis (with moderate loss, i.e., 25–50% of the implant height) since nonsurgical therapy, despite being conservative, has a high recurrence rate and typically does not resolve peri-implant disease [77,78,79,80]. The surgical treatment aims to decontaminate the implant surface, create a healthy hard and soft tissue peri-implant anatomy that allows easy cleaning, and regenerate the infrabony defect (if possible) [81,82]. Surgical approaches for treating peri-implantitis include open-flap debridement (OFD), apically positioned flap (APF), and guided bone regeneration (GBR). The surgical approach is often determined by the bone defect configuration, as the resective approach with APF (possibly with implantoplasty) is indicated for horizontal or one-wall defects, regenerative therapy is indicated for vertical two or three-wall defects, and combining approaches with combined defects.

#### 7.2.1. Open-Flap Debridement (OFD)

To cleanse the implant surfaces with OFD, a vestibular and lingually/palatally mucoperiosteal flap is elevated following the completion of the intracrevicolar incision. Then, inflamed tissue is degranulated, the implant surface is decontaminated mechanically or chemically, and the flap is repositioned with a suture [82]. OFD provides direct access to the defect, allowing for more effective implant decontaminating, and has a high implant survival rate and a moderate success rate (i.e., PD < 5 mm, absence of BOP and suppuration, preventing progressive bone loss) up to five years after treatment. According to Heitz-Mayfield et al. [82], this approach, combined with systemic antibiotic use, resulted in complete resolution in 53% of implants, and a reduction in inflammatory indices, but soft tissue recession of up to 1.8 mm after five years [58]. 

#### 7.2.2. Resective Technique and Apically Positioned Flap (APF)

Resective approaches aim to expose the supracrestal portion of the implant, eliminate pockets, and modify anatomy to improve oral hygiene practices. As such, it is indicated for supraosseous or intraosseous defects with one wall, whereas contraindicated in aesthetic areas. Decontamination of implant surfaces with APF occurs following flap elevation and degranulation of inflammatory tissue, the osteoplasty is performed under copious irrigation, and implantoplasty (i.e., removal of supracrestal threads with diamond burs and polishing of grommets under copious irrigation) may be added. According to studies by Carcuac et al. [83] and Serino et al. [84], treatment success is closely related to the initial severity of the pathology (i.e., a residual pocket > 6 mm would predict additional radiographic bone loss, OR = 7.4). Post-treatment resolution implants were more common in those with less initial bone loss (2–4 mm) than those with 5 mm bone loss (74% vs. 40%).

However, some evidence suggests that the resective approach combined with implantoplasty is superior to the resective technique alone. After three years, Romeo et al. [85] reported a 100% survival rate in the implantoplasty group versus 87.5% in the control group, as well as a progression of bone loss (1.44 mm mesially and 1.54 mm distally) in the control group, despite the same marginal bone loss after three years of implant surgery in the implantoplasty group [85]. However, implantoplasty is still controversial since it reduces the diameter and mechanical strength of the implant, increases the crown/implant ratio, and raises the risk of an implant or screw connection fracture [86]. Moreover, titanium particles released into surrounding tissues can promote inflammation and osteoclastic activity; thus, this approach is preferred in splint implants though not recommended for narrow implants.

#### 7.2.3. Regenerative Technique and Guided Bone Regeneration (GBR)

The regenerative approach aims to achieve reosteointegration by developing new osteogenesis on the previously decontaminated implant surface. Although the feasibility of attaining true reosteointegration on thoroughly decontaminated implants has been demonstrated histologically in animal research [87], histological evidence in humans is lacking [87]. However, BOP, CAL, and PD reductions, as well as radiographic filling of bone defects, can be clinically assessed, even if radiopacity of the bone defect does not always indicate successful osseointegration. The Eighth European Periodontology Workshop report highlighted that the success of regenerative procedures is strictly tied to a “proven method of decontamination of the implant surface”. However, no clinical, X-ray, or microbiological evidence exists to recommend a specific decontamination procedure currently; instead, a combination of physical and chemical decontamination approaches is recommended.

Although autologous bone is still considered the "gold standard" among grafting materials, several studies have revealed that it loses volume (40%) during the healing process compared to synthetic bone substitutes, which maintain their volume for years. According to Jepsen et al. [88], titanium granules provided the best radiographic filling of the bone defect (3.6 mm) and the best PD reduction (2.8 mm), together with bovine bone (3.1 mm), according to Aghazadeh et al. [89]. Concerning the usage of membranes, research has demonstrated that they improve clinical parameters (CAL and PD) compared to biomaterials alone; yet other studies found no statistically significant changes in defect reduction [20,86]. Membrane exposure in animal studies ranged from 13% to 38% and from 18% to 87.6% in clinical research, and early membrane exposure reduces the regenerative potential [90].

According to the meta-analysis by Chan et al. [90], the mean values of reduction of PD obtained with the various surgical approaches are:

- 2.38 ± 0.53 mm (37.9%) for OFD;
- 2.04 ± 0.15 mm (33.4%) for the resective surgery;
- 2.32 ± 1.29 mm (37.1%) for the use of bone substitutes;
- 3.16 ± 0.62 mm (48.2%) for regenerative procedures.

The most significant reduction in pocket depth was 5.4 mm, obtained using regenerative procedures [90], and around 2 mm of RBF (bone filling) for surgical procedures utilizing bone substitutes with or without membranes [90].

Compared to OFD, Toma et al. [91] found that reconstructive/regenerative surgery resulted in a 1.7 mm bone gain with 57% defect filling, whereas there was no difference in PD and BOP reduction. Reconstructive/regenerative therapy increased bone levels (2.0 mm) and CAL (1.8 mm) while decreasing PD (2.8 mm) and BOP [92].

### 7.3. Implant Removal

When there is osseointegration failure (severe loss, i.e., >50% of height), implant fractures, complicated implant designs (i.e., hollow-cylinder implant), or intricate infections affecting the surrounding anatomical structures (e.g., inferior alveolar nerve, maxillary sinus), the implant should be removed [93,94,95].

## 8. Conclusions

The prevalence of peri-implant diseases is constantly growing, particularly with the steady increase in using dental implants. Therefore, clinicians should adopt the new World Workshop definitions for more accessible communication and accurate diagnosis, as their application in epidemiological studies will help accurately estimate the incidence of peri-implant diseases. To effectively treat peri-implant diseases, risk factors should be first identified and minimized, supported by early diagnosis, patient inclusion in the maintenance protocol, and periodic clinical follow up with radiographic evaluations as necessary. Since several protocols are used for treating peri-implantitis, clinical success depends on the proper case evaluation. As such, it is recommended to combine different decontamination approaches since the effectiveness of surgical treatment is tied to a proven method of implant-surface decontamination; still, there is no clinical, radiological, or microbiological evidence indicating a specific protocol. Therefore, further randomized clinical trials investigating the different surgical approaches are required to address this knowledge gap and establish a predictable, efficient, and evidence-based approach for treating peri-implantitis.

## Figures and Tables

**Table 1:** Definitions of peri-implant disease cases according to the World Workshop on the Classification of Periodontal and Peri-implant diseases.

	Peri-Implant Health	Peri-Implant Mucositis	Peri-Implantitis (With rx and Clinical Records)	Peri-Implantitis (No rx and Clinical Records
Clinical signs of inflammation	−	+	+	+
BOP +/− suppuration	−	+	+	+
Increase in PD vs. previous visits	−	−	+	≥6 mm
Radiographic bone loss (except physiological remodeling)	−	−	+ physiological remodeling < 2 mm	≥3 mm

## Data Availability

All experimental data to support the findings of this study are available by contacting the corresponding author upon request.

## References

[1] Berglundh T., Persson L., Klinge B. (2002). A Systematic Review of the Incidence of Biological and Technical Complications in Implant Dentistry Reported in Prospective Longitudinal Studies of at Least 5 Years: Systematic Review of Implant Complications. J. Clin. Periodontol..

[2] Berglundh T., Armitage G., Araujo M.G., Avila-Ortiz G., Blanco J., Camargo P.M., Chen S., Cochran D., Derks J., Figuero E. (2018). Peri-Implant Diseases and Conditions: Consensus Report of Workgroup 4 of the 2017 World Workshop on the Classification of Periodontal and Peri-Implant Diseases and Conditions. J. Periodontol..

[3] Araujo M.G., Lindhe J. (2018). Peri-Implant Health. J. Periodontol..

[4] Renvert S., Persson G.R., Pirih F.Q., Camargo P.M. (2018). Peri-Implant Health, Peri-Implant Mucositis, and Peri-Implantitis: Case Definitions and Diagnostic Considerations. J. Clin. Periodontol..

[5] Tomasi C., Tessarolo F., Caola I., Piccoli F., Wennström J.L., Nollo G., Berglundh T. (2016). Early Healing of Peri-implant Mucosa in Man. J. Clin. Periodontol..

[6] Heitz-Mayfield L.J.A., Salvi G.E. (2018). Peri-Implant Mucositis. J. Clin. Periodontol..

[7] Pontoriero R., Tonelli M.P., Carnevale G., Mombelli A., Nyman S.R., Lang N.P. (1994). Experimentally Induced Peri-Implant Mucositis. A Clinical Study in Humans: Experimental Peri-Implant Mucositis. Clin. Oral Implant. Res..

[8] Meyer S., Giannopoulou C., Courvoisier D., Schimmel M., Müller F., Mombelli A. (2017). Experimental Mucositis and Experimental Gingivitis in Persons Aged 70 or over. Clinical and Biological Responses. Clin. Oral Implant. Res..

[9] Schwarz F., Derks J., Monje A., Wang H.-L. (2018). Peri-Implantitis. J. Periodontol..

[10] Berglundh T., Zitzmann N.U., Donati M. (2011). Are Peri-Implantitis Lesions Different from Periodontitis Lesions?: Peri-Implantitis and Periodontitis Lesions. J. Clin. Periodontol..

[11] Berglundh T., Gislason O., Lekholm U., Sennerby L., Lindhe J. (2004). Histopathological Observations of Human Periimplantitis Lesions. J. Clin. Periodontol..

[12] Derks J., Schaller D., Håkansson J., Wennström J.L., Tomasi C., Berglundh T. (2016). Peri-Implantitis—Onset and Pattern of Progression. J. Clin. Periodontol..

[13] Fransson C., Tomasi C., Pikner S.S., Gröndahl K., Wennström J.L., Leyland A.H., Berglundh T. (2010). Severity and Pattern of Peri-Implantitis-Associated Bone Loss. J. Clin. Periodontol..

[14] Fowkes F.G., Dobson A.J., Hensley M.J., Leeder S.R. (1984). The Role of Clinical Epidemiology in Medical Practice. Eff. Health Care.

[15] Derks J., Tomasi C. (2015). Peri-Implant Health and Disease. A Systematic Review of Current Epidemiology. J. Clin. Periodontol..

[16] Lee C.-T., Huang Y.-W., Zhu L., Weltman R. (2017). Prevalences of Peri-Implantitis and Peri-Implant Mucositis: Systematic Review and Meta-Analysis. J. Dent..

[17] Rakic M., Galindo-Moreno P., Monje A., Radovanovic S., Wang H.-L., Cochran D., Sculean A., Canullo L. (2018). How Frequent Does Peri-Implantitis Occur? A Systematic Review and Meta-Analysis. Clin. Oral Investig..

[18] Dreyer H., Grischke J., Tiede C., Eberhard J., Schweitzer A., Toikkanen S.E., Glöckner S., Krause G., Stiesch M. (2018). Epidemiology and Risk Factors of Peri-Implantitis: A Systematic Review. J. Periodontal Res..

[19] Cosgarea R., Sculean A., Shibli J.A., Salvi G.E. (2019). Prevalence of Peri-Implant Diseases—A Critical Review on the Current Evidence. Braz. Oral Res..

[20] Renvert S., Polyzois I., Claffey N. (2012). Surgical Therapy for the Control of Peri-Implantitis. Clin. Oral Implant. Res..

[21] Rinke S., Ohl S., Ziebolz D., Lange K., Eickholz P. (2011). Prevalence of Periimplant Disease in Partially Edentulous Patients: A Practice-Based Cross-Sectional Study. Clin. Oral Implant. Res..

[22] Roos-Jansåker A.M., Lindahl C., Renvert H., Renvert S. (2006). Nine- to Fourteen-Year Follow-up of Implant Treatment. Part I: Implant Loss and Associations to Various Factors. J. Clin. Periodontol..

[23] Monje A., Catena A., Borgnakke W.S. (2017). Association between Diabetes Mellitus/Hyperglycaemia and Peri-Implant Diseases: Systematic Review and Meta-Analysis. J. Clin. Periodontol..

[24] Roccuzzo M., De Angelis N., Bonino L., Aglietta M. (2010). Ten-Year Results of a Three-Arm Prospective Cohort Study on Implants in Periodontally Compromised Patients. Part 1: Implant Loss and Radiographic Bone Loss. Clin. Oral Implant. Res..

[25] Serino G., Ström C. (2009). Peri-Implantitis in Partially Edentulous Patients: Association with Inadequate Plaque Control. Clin. Oral Implant. Res..

[26] Staubli N., Walter C., Schmidt J.C., Weiger R., Zitzmann N.U. (2017). Excess Cement and the Risk of Peri-Implant Disease—A Systematic Review. Clin. Oral Impl. Res..

[27] Scarano A., Inchingolo F., Scogna S., Leo L., Greco Lucchina A., Mavriqi L. (2021). Peri-Implant Disease Caused by Residual Cement around Implant-Supported Restorations: A Clinical Report. J. Biol. Regul. Homeost. Agents.

[28] Costa F.O., Takenaka-Martinez S., Cota L.O.M., Ferreira S.D., Silva G.L.M., Costa J.E. (2012). Peri-Implant Disease in Subjects with and without Preventive Maintenance: A 5-Year Follow-Up. J. Clin. Periodontol..

[29] Lin G.-H., Chan H.-L., Wang H.-L. (2013). The Significance of Keratinized Mucosa on Implant Health: A Systematic Review. J. Periodontol..

[30] Gobbato L., Avila-Ortiz G., Sohrabi K., Wang C.-W., Karimbux N. (2013). The Effect of Keratinized Mucosa Width on Peri-Implant Health: A Systematic Review. Int. J. Oral Maxillofac. Implant..

[31] Wilson T.G., Valderrama P., Burbano M., Blansett J., Levine R., Kessler H., Rodrigues D.C. (2015). Foreign Bodies Associated with Peri-Implantitis Human Biopsies. J. Periodontol..

[32] Noumbissi S., Scarano A., Gupta S. (2019). A Literature Review Study on Atomic Ions Dissolution of Titanium and Its Alloys in Implant Dentistry. Materials.

[33] Daubert D., Pozhitkov A., McLean J., Kotsakis G. (2018). Titanium as a Modifier of the Peri-Implant Microbiome Structure. Clin. Implant Dent. Relat. Res..

[34] Hashim D., Cionca N. (2020). A Comprehensive Review of Peri-Implantitis Risk Factors. Curr. Oral Health Rep..

[35] Sasada Y., Cochran D.L. (2017). Implant-Abutment Connections: A Review of Biologic Consequences and Peri-Implantitis Implications. Int. J. Oral Maxillofac. Implant..

[36] Carinci F., Lauritano D., Bignozzi C.A., Pazzi D., Candotto V., Santos de Oliveira P., Scarano A. (2019). A New Strategy Against Peri-Implantitis: Antibacterial Internal Coating. Int. J. Mol. Sci..

[37] Scarano A., de Oliveira P.S., Leo L., Festa F., Carinci F., Lorusso F. (2021). Evaluation of a New Antibacterial Coating of the Internal Chamber of an Implant via Real Time Measurement of Volatile Organic Compounds (VOCs). Front. Biosci..

[38] Sanz M., Chapple I.L., on behalf of Working Group 4 of the VIII European Workshop on Periodontology (2012). Clinical Research on Peri-Implant Diseases: Consensus Report of Working Group 4. J. Clin. Periodontol..

[39] Shibli J.A., Melo L., Ferrari D.S., Figueiredo L.C., Faveri M., Feres M. (2008). Composition of Supra- and Subgingival Biofilm of Subjects with Healthy and Diseased Implants. Clin. Oral Implant. Res..

[40] Socransky S.S., Haffajee A.D. (2005). Periodontal Microbial Ecology. Periodontol. 2000.

[41] Albertini M., López-Cerero L., O’Sullivan M.G., Chereguini C.F., Ballesta S., Ríos V., Herrero-Climent M., Bullón P. (2015). Assessment of Periodontal and Opportunistic Flora in Patients with Peri-Implantitis. Clin. Oral Implant. Res..

[42] Kim H.-J., Ahn D.-H., Yu Y., Han H., Kim S.Y., Joo J.-Y., Chung J., Na H.S., Lee J.-Y. (2023). Microbial Profiling of Peri-Implantitis Compared to the Periodontal Microbiota in Health and Disease Using 16S RRNA Sequencing. J. Periodontal Implant Sci..

[43] Kensara A., Saito H., Mongodin E.F., Masri R. (2023). Microbiological Profile of Peri-implantitis: Analyses of Microbiome within Dental Implants. J. Prosthodont..

[44] Lafaurie G.I., Sabogal M.A., Castillo D.M., Rincón M.V., Gómez L.A., Lesmes Y.A., Chambrone L. (2017). Microbiome and Microbial Biofilm Profiles of Peri-Implantitis: A Systematic Review. J. Periodontol..

[45] Persson G.R., Renvert S. (2014). Cluster of Bacteria Associated with Peri-Implantitis: Pathogens in Peri-Implantitis. Clin. Implant Dent. Relat. Res..

[46] Schwarz F., Becker K., Rahn S., Hegewald A., Pfeffer K., Henrich B. (2015). Real-Time PCR Analysis of Fungal Organisms and Bacterial Species at Peri-Implantitis Sites. Int. J. Implant Dent..

[47] Jankovic S., Aleksic Z., Dimitrijevic B., Lekovic V., Camargo P., Kenney B. (2011). Prevalence of Human Cytomegalovirus and Epstein-Barr Virus in Subgingival Plaque at Peri-Implantitis, Mucositis and Healthy Sites. A Pilot Study. Int. J. Oral Maxillofac. Surg..

[48] Jepsen S., Berglundh T., Genco R., Aass A.M., Demirel K., Derks J., Figuero E., Giovannoli J.L., Goldstein M., Lambert F. (2015). Primary Prevention of Peri-Implantitis: Managing Peri-Implant Mucositis. J. Clin. Periodontol..

[49] Tonetti M.S., Chapple I.L.C., Jepsen S., Sanz M. (2015). Primary and Secondary Prevention of Periodontal and Peri-Implant Diseases: Introduction to, and Objectives of the 11 th European Workshop on Periodontology Consensus Conference. J. Clin. Periodontol..

[50] Sinjab K., Garaicoa-Pazmino C., Wang H.-L. (2018). Decision Making for Management of Periimplant Diseases. Implant Dent..

[51] Kormas I., Pedercini C., Pedercini A., Raptopoulos M., Alassy H., Wolff L.F. (2020). Peri-Implant Diseases: Diagnosis, Clinical, Histological, Microbiological Characteristics and Treatment Strategies. A Narrative Review. Antibiotics.

[52] Figuero E., Graziani F., Sanz I., Herrera D., Sanz M. (2014). Management of Peri-Implant Mucositis and Peri-Implantitis. Periodontol. 2000.

[53] Wilson T.G., Valderrama P., Rodrigues D.B.C. (2014). Commentary: The Case for Routine Maintenance of Dental Implants. J. Periodontol..

[54] Schmidt K.E., Auschill T.M., Heumann C., Frankenberger R., Eick S., Sculean A., Arweiler N.B. (2017). Influence of Different Instrumentation Modalities on the Surface Characteristics and Biofilm Formation on Dental Implant Neck, *In Vitro*. Clin. Oral Implant. Res..

[55] Renvert S., Samuelsson E., Lindahl C., Persson G.R. (2009). Mechanical Non-Surgical Treatment of Peri-Implantitis: A Double-Blind Randomized Longitudinal Clinical Study. I: Clinical Results. J. Clin. Periodontol..

[56] Sirinirund B., Garaicoa-Pazmino C., Wang H.-L. (2019). Effects of Mechanical Instrumentation with Commercially Available Instruments Used in Supportive Peri-Implant Therapy: An In Vitro Study. Int. J. Oral Maxillofac. Implant..

[57] Tastepe C.S., van Waas R., Liu Y., Wismeijer D. (2012). Air Powder Abrasive Treatment as an Implant Surface Cleaning Method: A Literature Review. Int. J. Oral Maxillofac. Implant..

[58] Schwarz F., Becker K., Renvert S. (2015). Efficacy of Air Polishing for the Non-Surgical Treatment of Peri-Implant Diseases: A Systematic Review. J. Clin. Periodontol..

[59] Pisano M., Amato A., Sammartino P., Iandolo A., Martina S., Caggiano M. (2021). Laser Therapy in the Treatment of Peri-Implantitis: State-of-the-Art, Literature Review and Meta-Analysis. Appl. Sci..

[60] Renvert S., Lindahl C., Roos Jansåker A.-M., Persson G.R. (2011). Treatment of Peri-Implantitis Using an Er:YAG Laser or an Air-Abrasive Device: A Randomized Clinical Trial: Non-Surgical Treatment of Peri-Implantitis. J. Clin. Periodontol..

[61] Schwarz F., Bieling K., Bonsmann M., Latz T., Becker J. (2006). Nonsurgical Treatment of Moderate and Advanced Periimplantitis Lesions: A Controlled Clinical Study. Clin. Oral Investig..

[62] Ramanauskaite A., Schwarz F., Cafferata E.A., Sahrmann P. (2023). Photo/Mechanical and Physical Implant Surface Decontamination Approaches in Conjunction with Surgical Peri-Implantitis Treatment: A Systematic Review. J. Clin. Periodontol..

[63] Sivaramakrishnan G., Sridharan K. (2018). Photodynamic Therapy for the Treatment of Peri-Implant Diseases: A Network Meta-Analysis of Randomized Controlled Trials. Photodiagn. Photodyn. Ther..

[64] Garaicoa-Pazmino C., Sinjab K., Wang H.-L. (2019). Current Protocols for the Treatment of Peri-Implantitis. Curr. Oral Health Rep..

[65] Dommisch H., Hoedke D., Valles C., Vilarrasa J., Jepsen S., Pascual La Rocca A. (2022). Efficacy of Professionally Administered Chemical Agents as an Adjunctive Treatment to Sub-marginal Instrumentation during the Therapy of Peri-implant Mucositis. J. Clin. Periodontol..

[66] Wilensky A., Shapira L., Limones A., Martin C. (2023). The Efficacy of Implant Surface Decontamination Using Chemicals during Surgical Treatment of Peri-Implantitis: A Systematic Review and Meta-Analysis. J. Clin. Periodontol..

[67] Galofré M., Palao D., Vicario M., Nart J., Violant D. (2018). Clinical and Microbiological Evaluation of the Effect of *Lactobacillus Reuteri* in the Treatment of Mucositis and Peri-Implantitis: A Triple-Blind Randomized Clinical Trial. J. Periodont. Res..

[68] Hallström H., Persson G.R., Lindgren S., Olofsson M., Renvert S. (2012). Systemic Antibiotics and Debridement of Peri-Implant Mucositis. A Randomized Clinical Trial. J. Clin. Periodontol..

[69] Pulcini A., Bollaín J., Sanz-Sánchez I., Figuero E., Alonso B., Sanz M., Herrera D. (2019). Clinical Effects of the Adjunctive Use of a 0.03% Chlorhexidine and 0.05% Cetylpyridinium Chloride Mouth Rinse in the Management of Peri-Implant Diseases: A Randomized Clinical Trial. J. Clin. Periodontol..

[70] Gennai S., Bollain J., Ambrosio N., Marruganti C., Graziani F., Figuero E. (2023). Efficacy of Adjunctive Measures in Peri-implant Mucositis. A Systematic Review and Meta-analysis. J. Clin. Periodontol..

[71] Teughels W., Seyssens L., Christiaens V., Temmerman A., Castro A.B., Cosyn J. (2023). Adjunctive Locally and Systemically Delivered Antimicrobials during Surgical Treatment of Peri-implantitis: A Systematic Review. J. Clin. Periodontol..

[72] Verdugo F. (2018). Risk of Superinfection in Peri-Implantitis After Systemic Broad Spectrum Antibiotics. Int. J. Periodont. Restor. Dent..

[73] Koch F.P., Kaemmerer P.W., Biesterfeld S., Kunkel M., Wagner W. (2011). Effectiveness of Autofluorescence to Identify Suspicious Oral Lesions—A Prospective, Blinded Clinical Trial. Clin. Oral Investig..

[74] Schneider S., Rudolph M., Bause V., Terfort A. (2018). Electrochemical Removal of Biofilms from Titanium Dental Implant Surfaces. Bioelectrochemistry.

[75] Dhaliwal J.S., Abd Rahman N.A., Ming L.C., Dhaliwal S.K.S., Knights J., Albuquerque Junior R.F. (2021). Microbial Biofilm Decontamination on Dental Implant Surfaces: A Mini Review. Front. Cell. Infect. Microbiol..

[76] Roccuzzo A., De Ry S.P., Sculean A., Roccuzzo M., Salvi G.E. (2020). Current Approaches for the Non-Surgical Management of Peri-Implant Diseases. Curr. Oral Health Rep..

[77] Subramani K., Wismeijer D. (2012). Decontamination of Titanium Implant Surface and Re-Osseointegration to Treat Peri-Implantitis: A Literature Review. Int. J. Oral Maxillofac. Implant..

[78] Lindhe J., Meyle J., on behalf of Group D of the European Workshop on Periodontology (2008). Peri-Implant Diseases: Consensus Report of the Sixth European Workshop on Periodontology. J. Clin. Periodontol..

[79] Ramanauskaite A., Fretwurst T., Schwarz F. (2021). Efficacy of Alternative or Adjunctive Measures to Conventional Non-Surgical and Surgical Treatment of Peri-Implant Mucositis and Peri-Implantitis: A Systematic Review and Meta-Analysis. Int. J. Implant Dent..

[80] Suárez-López del Amo F., Yu S.-H., Wang H.-L. (2016). Non-Surgical Therapy for Peri-Implant Diseases: A Systematic Review. J. Oral Maxillofac. Res..

[81] Renvert S., Polyzois I., Maguire R. (2009). Re-Osseointegration on Previously Contaminated Surfaces: A Systematic Review. Clin. Oral Implant. Res..

[82] Heitz-Mayfield L.J.A., Salvi G.E., Mombelli A., Faddy M., Lang N.P., On behalf of the Implant Complication Research Group Anti-Infective Surgical Therapy of Peri-Implantitis (2012). A 12-Month Prospective Clinical Study. Clin. Oral Implant. Res..

[83] Carcuac O., Derks J., Abrahamsson I., Wennström J.L., Berglundh T. (2020). Risk for Recurrence of Disease Following Surgical Therapy of Peri-Implantitis—A Prospective Longitudinal Study. Clin. Oral Implant. Res..

[84] Serino G., Turri A. (2011). Outcome of Surgical Treatment of Peri-Implantitis: Results from a 2-Year Prospective Clinical Study in Humans: Outcome of Surgical Treatment of Peri-Implantitis. Clin. Oral Implant. Res..

[85] Romeo E., Lops D., Chiapasco M., Ghisolfi M., Vogel G. (2007). Therapy of Peri-Implantitis with Resective Surgery. A 3-Year Clinical Trial on Rough Screw-Shaped Oral Implants. Part II: Radiographic Outcome. Clin. Oral Implant. Res..

[86] Aljohani M., Yong S.L., Bin Rahmah A. (2020). The Effect of Surgical Regenerative Treatment for Peri-Implantitis: A Systematic Review. Saudi Dent. J..

[87] Koo K.-T., Khoury F., Keeve P.L., Schwarz F., Ramanauskaite A., Sculean A., Romanos G. (2019). Implant Surface Decontamination by Surgical Treatment of Periimplantitis: A Literature Review. Implant Dent..

[88] Jepsen K., Jepsen S., Laine M.L., Anssari Moin D., Pilloni A., Zeza B., Sanz M., Ortiz-Vigon A., Roos-Jansåker A.M., Renvert S. (2016). Reconstruction of Peri-Implant Osseous Defects: A Multicenter Randomized Trial. J. Dent. Res..

[89] Aghazadeh A., Persson R.G., Renvert S. (2020). Impact of Bone Defect Morphology on the Outcome of Reconstructive Treatment of Peri-Implantitis. Int. J. Implant Dent..

[90] Chan H.-L., Lin G.-H., Suarez F., MacEachern M., Wang H.-L. (2014). Surgical Management of Peri-Implantitis: A Systematic Review and Meta-Analysis of Treatment Outcomes. J. Periodontol..

[91] Toma S., Brecx M.C., Lasserre J.F. (2019). Clinical Evaluation of Three Surgical Modalities in the Treatment of Peri-Implantitis: A Randomized Controlled Clinical Trial. J. Clin. Med..

[92] Tomasi C., Regidor E., Ortiz-Vigón A., Derks J. (2019). Efficacy of Reconstructive Surgical Therapy at Peri-Implantitis-Related Bone Defects. A Systematic Review and Meta-Analysis. J. Clin. Periodontol..

[93] Schwarz F., Ramanauskaite A. (2022). It Is All about Peri-implant Tissue Health. Periodontol. 2000.

[94] Scarano A., Barros R.R.M., Iezzi G., Piattelli A., Novaes A.B. (2009). Acellular Dermal Matrix Graft for Gingival Augmentation: A Preliminary Clinical, Histologic, and Ultrastructural Evaluation. J. Periodontol..

[95] Scarano A., Piattelli A., Polimeni A., Di Iorio D., Carinci F. (2010). Bacterial Adhesion on Commercially Pure Titanium and Anatase-Coated Titanium Healing Screws: An In Vivo Human Study. J. Periodontol..

